# pH-Responsive Au(i)-disulfide nanoparticles with tunable aggregation-induced emission for monitoring intragastric acidity†

**DOI:** 10.1039/d0sc01843k

**Published:** 2020-04-24

**Authors:** Jianxing Wang, Jie Li, Ying Li, Zhijun Zhang, Lei Wang, Dong Wang, Lei Su, Xueji Zhang, Ben Zhong Tang

**Affiliations:** Center for AIE Research, College of Materials Science and Engineering, Shenzhen University Shenzhen 518060 China wangd@szu.edu.cn; College of Physics and Optoelectronic Engineering, Shenzhen University Shenzhen 518060 China; Research Center for Bioengineering and Sensing Technology, School of Chemistry and Biological Engineering, University of Science and Technology Beijing Beijing 100083 China sulei@ustb.edu.cn; School of Biomedical Engineering, Shenzhen University Health Science Center Shenzhen 518060 China zhangxueji@ustb.edu.cn; Hong Kong Branch of Chinese National Engineering Research Center for Tissue Restoration and Reconstruction, Department of Chemistry, The Hong Kong University of Science and Technology Clear Water Bay Kowloon Hong Kong China tangbenz@ust.hk

## Abstract

Aggregation-induced emission (AIE)-featuring Au(i) complexes are superior probes for physiological environment monitoring in living organisms owing to their excellent biocompatibility and efficient luminescent properties. However, the intrinsic obstacle of poor water stability and lack of response to biological stimuli greatly restrict their practical application in biological systems. Herein, water-stable and pH-responsive Au(i)-disulfide nanoparticles (NPs) with AIE characteristics were designed. The NPs were prepared by integrating a pH-responsive moiety, cysteine (Cys), into Au(i)-thiolate (SR) complexes, and the Au(i)-SR-Cys structure was formed through disulfide bonds. Hydrophilic Cys was located on the outer layer of the NPs, endowing the spherical NPs with high stability and remarkable monodispersity in water. In addition, Cys endowed the NPs with pH-responsive characteristics. These unique advantages enable them to hold great promise as luminescent probes to monitor intragastric acidity in an acid suppression therapy. To the best of our knowledge, this work is the first example of luminescent Au(i) materials to monitor physiological changes.

## Abstract 2

The water-stable and pH-responsive Au(i)-disulfide NPs serve as a luminescent probe for monitoring intragastric acidity in an acid-suppressed therapy.

## Graphical abstract

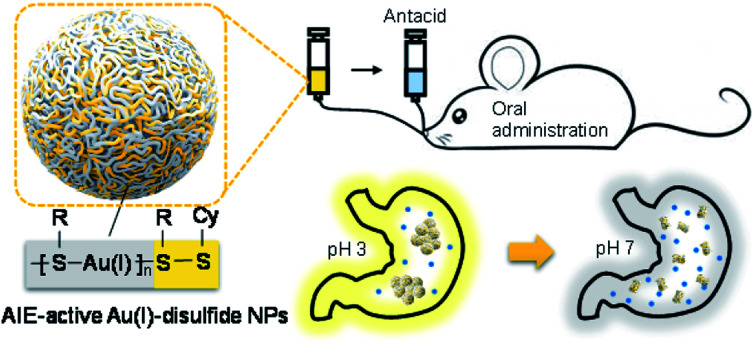

## Introduction

In pathophysiology, most human diseases are related to an abnormal physiological environment, which has always been considered as a clinical sign of these diseases.^1–3^ Monitoring the abnormal physiological environment could provide comprehensive and deep insights into disease diagnosis, as well as the applications of tracking and evaluating the therapeutic effect.^4^ Taking the treatment of gastrointestinal diseases as examples, intragastric acidity always needs to be suppressed to a certain degree in a certain time, for safeguarding the activity of therapeutic drugs or avoiding creation of mucosal lesions in peptic ulcers.^5–8^ Monitoring the intragastric acidity in the acid-suppressed therapy can precisely improve the therapeutic effect. Fluorescence techniques have been widely used in monitoring physiological changes, owing to the advantages of simple operation, high sensitivity and excellent spatial–temporal resolution.^9–11^ However, most luminescent dyes, such as traditional organic fluorophores, possess intrinsic obstacles of poor biocompatibility and aggregation-caused quenching (ACQ) effect, which greatly restrict them acting as luminescent probes to monitor physiological changes. Therefore, it is highly desirable to develop fluorescent probes with excellent biocompatibility and high-performance emission to monitor physiological changes in biological systems.

As a family of luminescent probes with excellent biocompatibility, gold-based luminescent materials have been widely applied in biological systems,^12–14^ Of particular interest are aggregation-induced emission (AIE)-featuring Au(i) complexes.^15–17^ The AIE characteristics enable Au(i) materials to overcome the difficulties of the ACQ effect, making them serving as superior probes to monitor physiological changes in biological systems, by virtue of their excellent sensitivity, large Stokes shift, high signal-to noise ratio and high photostability.^18–22^ However, scarcely any of AIE-featuring Au(i) complexes are applied in living systems, since most of them showed poor stability in water, large size, and a strong tendency to precipitate from water, making them difficult to survive in physiological environments.^15,23,24^ Additionally, only a handful of AIE-featuring Au(i) complexes exhibited specific responses to biological environments resulting from the lack of well-designed responsive groups.^25,26^ Therefore, to specifically monitor physiological changes in living organisms, it is of great significance to manufacture water-stable and stimulus-responsive AIE-featuring Au(i) complexes.

**Scheme 1:**
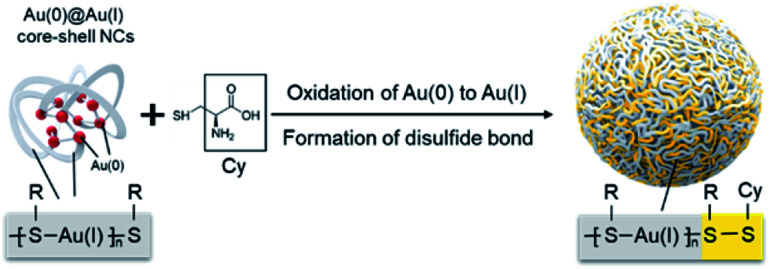
Schematic illustration of the preparation of Au(i)-disulfide NPs.

Integrating hydrophilic responsive fragments into Au(i) complexes has great potential in addressing this issue. The excellent hydrophilic properties could improve the water stability of Au(i) complexes, meanwhile the responsive merit is able to endow them with a highly specific response to physiological environments. Based on this assumption, herein, we presented a stable and pH-responsive Au(i) complex where cysteine (Cys), a natural thiol-based amino acid, was hereby selected to be introduced into the Au(i)-thiolate (SR) complex by means of oxidizing Au(0) with the thiol groups under mild reaction conditions. The obtained Au(i) complex exhibited intense AIE effects. Moreover, the carboxyl and amino groups of Cys possess the unique capacity to change the charged states over pH conditions, which further varied the molecular arrangement of the Au(i) complex and altered the luminescent emission. Most interestingly, a specific building unit of Au(i)-SR-Cys, other than Au(i)-SR, was formed during the oxidation of Au(i)@Au(0) core–shell nanoclusters (NCs) (Scheme 1). Owing to the pH-responsive AIE characteristics, the Au(i)-disulfide complex-based nanoparticles (NPs) can serve as a prominent luminescent probe to determine intragastric acidity during acid suppression therapy.

## Results and discussion

**Fig. 1:**
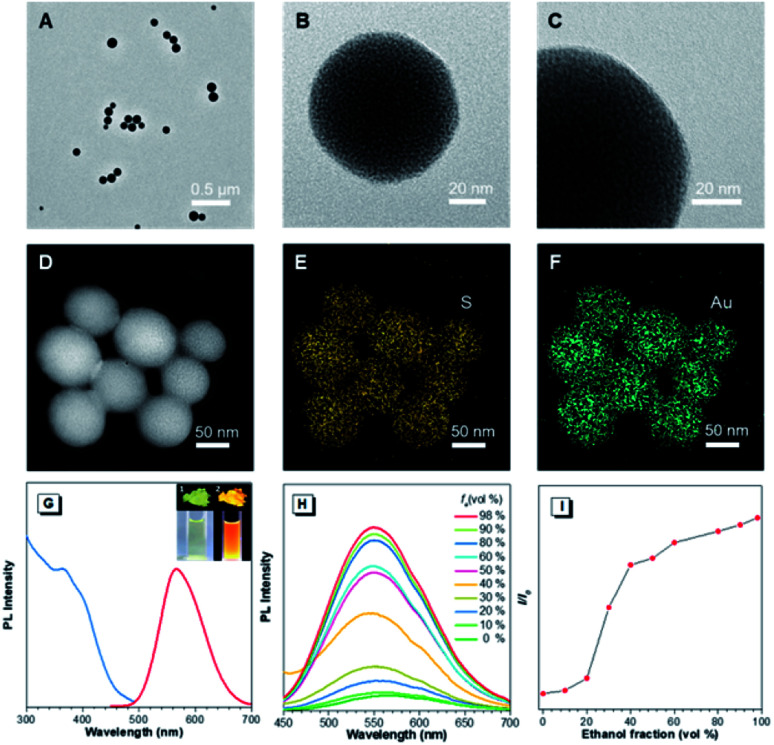
Characterization experiments of Au(i)-disulfide NPs. (A–C) TEM images of Au(i)-disulfide NPs at different magnifications. (D–F) STEM-EDS mapping images of Au(i)-disulfide NPs. (G) Photoluminescence (red line, *λ*_ex_ = 365 nm) and photoexcitation (blue line, *λ*_em_ = 565 nm) spectra of Au(i)-disulfide NPs. (insets) Digital photos of Au(i)-disulfide NPs in the solid state (top row) and in water (bottom row) under (1) visible light and (2) UV light. (H) Photoluminescence spectra of Au(i)-disulfide NPs in a mixture of water–ethanol with different ethanol fractions (*f*_e_), *λ*_ex_ = 365 nm and (I) variation in *I*/*I*_0_ with *f*_e_, *λ*_em_ = 565 nm.

In the preliminary study, the Au(i)@Au(0) core–shell NCs were prepared *via* the reduction of Au(iii) salts by a mild reductant (glutathione, GSH). The TEM images showed that the NCs were small clusters with a size of less than 2 nm (Fig. S1†). The Au 4f spectrum of the NCs could be deconvoluted into Au(0) and Au(i) components with binding energies (BEs) of 83.7 eV and 84.4 eV corresponding to the inner Au(0) core and the outer Au(i) shell, respectively, and the Au(i) content in all the Au atoms was determined to be 75% (Fig. S2A†). Then, the pre-synthesized NCs and Cys were incubated in water at 55 °C for an hour, facilely producing Cys-contained Au(i) complex-based NPs. The XPS spectrum of the reactants only exhibited one peak with a BE of 84.4 eV, suggesting that all Au(0) atoms have been oxidized to the Au(i) state (Fig. S2B†). As illustrated in Fig. 1A–C, well-defined spherical NPs with an average size of ∼110 nm were presented, which were much larger than NCs (<2 nm). The size of NPs basically coincided with the DLS result (Fig. S3†). Moreover, a homogeneous distribution of elements Au and S was found, indicating that these spherical NPs were composed of the as-synthesized Au(i) complex (Fig. 1D–F). The complex exhibited a much stronger Rayleigh scattering by a red laser point compared to the NC solution (Fig. S4†), which confirmed the formation of large Au(i) NPs as well. The large size of Au(i) NPs also led to a hyperchromic shift in the UV-vis absorption spectrum compared with NCs, resulting from the increase of background scattering (Fig. S5†). The Au(i) NPs emitted orange luminescence both in solution and solid states under UV irradiation (insets of Fig. 1G). The emission maxima of the Au(i) NPs peaked at 565 nm (Fig. 1G, red line), exhibiting 6.2% of quantum yield in water with fluorescein as the reference. In addition, the fluorescence intensity of the Au(i) NPs remained almost constant during storage at room temperature for a week (Fig. S6†), revealing the unique advantage in terms of photostability. The Au(i) NPs exhibited a broad excitation with a shoulder peak at 365 nm (Fig. 1G, blue line), which suggested a large Stokes shift (>200 nm). Moreover, it was observed that the Au(i) NPs were phosphorescent, which was verified by the PL lifetime measurements (Fig. S7†). The luminescence of Au(i) NPs could be attributed to the slower radiative relaxation *via* the triplet-centered states through ligand-to-metal charge transfer (LMCT) or ligand-to-metal-metal charge transfer (LMMCT).

**Scheme 2:**
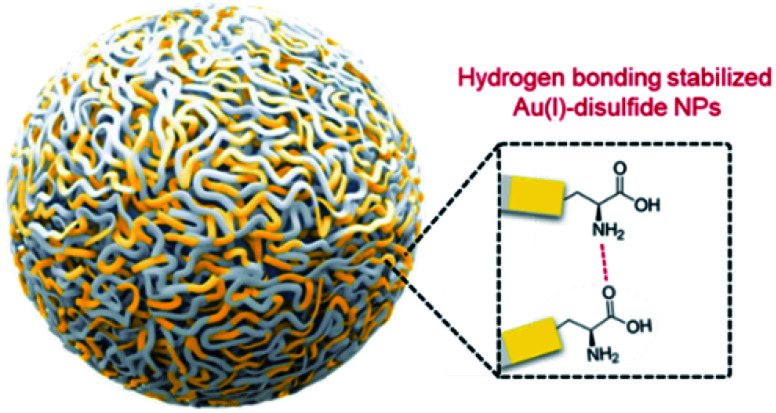
Schematic illustration of the structure of Au(i)-disulfide NPs. The hydrogen bonds between Cys existed at the out layer of Au(i)-disulfide NPs, which maintain the structural stability of NPs in water.

As confirmed, the as-synthesized Au(i) complex could spontaneously form luminescent spherical NPs in water. The structure of the obtained Au(i) NPs was further studied. The HR-ESI-MS spectrum of the Au(i) NPs indicated that the main composition was Au_2_S_3_Cys (Fig. S8†). The S fragments in Au_2_S_3_Cys originated from GSH or Cys, owing to the cleavage of the sulfur–carbon (S–C) bond in the ionization process during ESI-MS detection.^27^ The ratio of Au(i) and thiolates (SR) is usually 1 : 1 in a typical Au(i)-SR complex, denoted as [Au(i)-SR]_*n*_.^23,28^ The abnormal ratio of Au(i) and SR in Au_2_S_3_Cys could be consequently attributed to the potential existence of the Au(i)-disulfide complex in the Au(i) NPs. The Fourier transform infrared (FTIR) spectra revealed the absence of the S–H band at 2550 cm^−1^ and the appearance of a new band assigned to the S–S stretching vibration at 660 cm^−1^ (Fig. S9†),^29,30^ solidly suggesting the formation of disulfide bonds in the Au(i) complex. To further confirm the presence of disulfide bonds, tris(2-carboxyethyl)phosphine (TCEP), which is capable of breaking down disulfide bonds, was added to the Au(i) NP solution. Only loose and disordered morphologies could be observed in the TEM images, strongly revealing that the well-defined Au(i) nanospheres disintegrated after the cleavage of the disulfide bonds (Fig. S10A†). The UV-vis absorption spectra showed that the shoulder peak at 400 nm disappeared and no background scattering at wavelength >400 nm was found, reiterating the disintegration of Au(i) NPs (Fig. S10B†). Correspondingly, the emission of Au(i) NPs was quenched after addition of TCEP (Fig. S10C†), which further verified the disintegration of Au(i) NPs. It seems reasonable to infer that the disulfide bonds existed in the Au(i) NPs and played an indispensable role in maintaining their spherical nanostructure. Additionally, the presence of hydrogen bonds in Au(i)-disulfide NPs was further explored. Compared with Au(0)@Au(i) core–shell NCs, the FTIR spectrum of Au(i)-disulfide NPs exhibited a new broad band at 3286–3588 cm^−1^ (Fig. S9†), which was indexed to the hydrogen bonded (O–H) stretching vibrations from the carboxylic groups of Cys.^31^ To break down these hydrogen bonds, ethanol was added into the solution of Au(i)-disulfide NPs. Interestingly, these NPs formed large aggregates with a hydrodynamic diameter of ∼2.1 μm in a water/ethanol mixture with the ethanol fraction (*f*_e_) of 70% (Fig. S11†). The hydrogen bonds between Cys must exist at the outer layer of Au(i)-disulfide NPs, otherwise destroying the hydrogen bonds would lead to the disintegration of these NPs. The structure of Au(i)-disulfide NPs is illustrated in Scheme 2. Moreover, by increasing ethanol fraction, the PL intensity of the Au(i) NPs increased monotonically with their rising aggregation extent (Fig. 1H and I), demonstrating typical AIE characteristics.

**Fig. 2:**
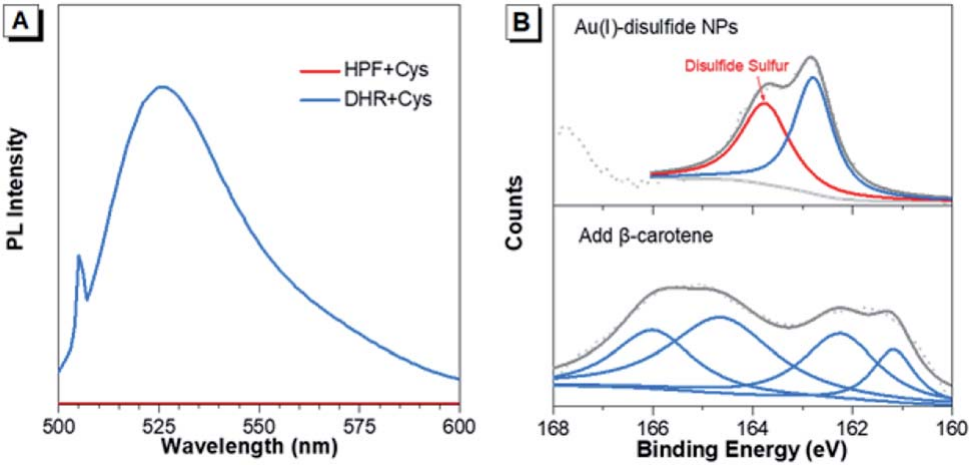
The formation mechanism of disulfide bonds. (A) PL spectra of the reaction solution after incubation of the fresh Cys aqueous solution with HPF (red line) and DHR (blue line) respectively at 55 °C for 1 hour. (B) S2p XPS spectra of Au(i)-disulfide NPs (top) and the reaction product after adding β-carotene in the synthesis of Au(i)-disulfide NPs (bottom).

Furthermore, the formation mechanism of disulfide bonds in Au(i) NPs was explored. Under the same reaction conditions as the synthesis of Au(i) NPs, two commonly used ROS indicators, dihydrorhodamine123 (DHR123) and hydroxyphenyl fluorescein (HPF) were respectively mixed with the fresh Cys aqueous solution. DHR123 and HPF are originally nonemissive in solution, while their luminescence can be rapidly triggered by superoxide radicals and hydroxyl radicals, respectively. Intriguingly, the green luminescence of DHR123 was rapidly boosted after the addition (Fig. 2A). This indicated that superoxide radicals spontaneously generated in the Cys solution in the synthesis process of Au(i)-disulfide NPs. The superoxide radicals have been reported to be a necessary component to the thiol-induced oxidation of Au(0) cores in Au NCs.^32^ Additionally, thiol has been reported to be capable of giving a proton to O_2_ to generate a superoxide radical and a thiyl radical.^32,33^ It was reasonable to conjecture that thiyl radicals were also produced upon the formation of Au(i)-disulfide NPs as well. To prove the existence of thiyl radicals, excessive β-carotene, a thiyl radical scavenger,^34^ was added into the reaction mixtures for the synthesis of Au(i)-disulfide NPs. Interestingly, the S2p spectra demonstrated that the peak at 163–164 eV which was attributed to the disulfide sulfur of Au(i) NPs disappeared in the spectrum of the reaction products (Fig. 2B).^35^ These results revealed the existence of thiyl radicals in the synthesis process, which played an indispensable role in the formation of disulfide bonds in Au(i) NPs. We hereby propose a radical-based mechanism for the formation of disulfide bonds in Au(i) NPs (Fig. S12†). Briefly, S–Au bond on the surface of Au(0)@Au(i) core–shell NCs is cleaved by the thiyl radical of Cys to expose the sulfur atom of Au(I)-SR complexes where the thiyl radical would covalently bind to it and form disulfide bond. Then the Au(i)-disulfide complexes would be released into the solution after the oxidation of Au(0) by superoxide radicals. This cycle can repeat, completely oxidizing all the Au(0) atoms to Au(i) atoms in the formation of Au(i)-disulfide complexes.

**Fig. 3:**
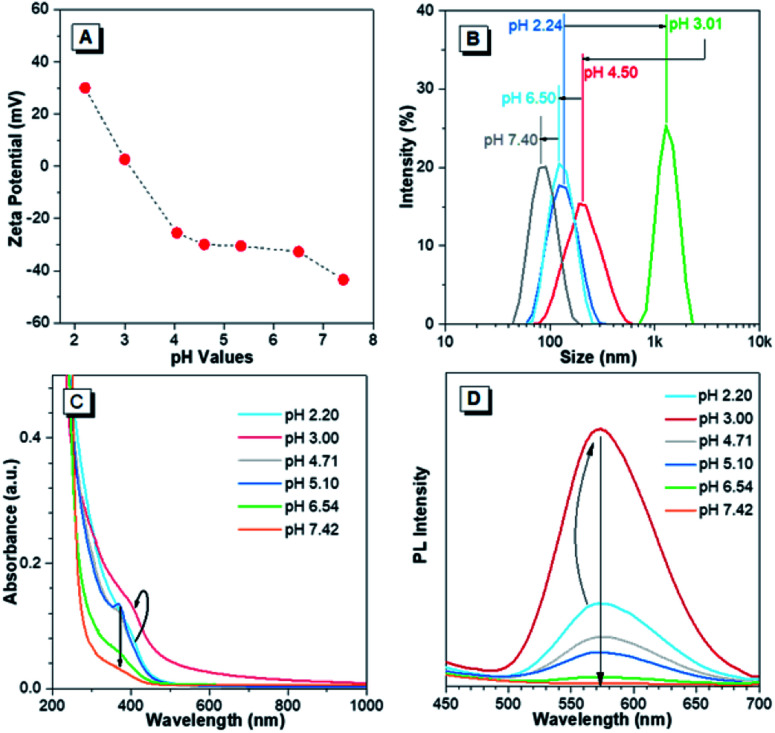
pH-Responsive characteristics of Au(i)-disulfide NPs. (A) Zeta-potential of Au(i)-disulfide NPs in response to solution pH values. (B) Hydrodynamic diameter (measured by dynamic light scattering) of Au(i)-disulfide NPs in response to solution pH values. (C) pH-Dependent UV-vis absorption spectra of Au(i)-disulfide NPs. (D) pH-Dependent photoemission spectra of the Au(i)-disulfide NPs. The arrows in (C) and (D) respectively represent the PL and UV-absorption fluctuation of Au(i)-disulfide NPs in response to the pH change.

The pH-responsive characteristics of Au(i)-disulfide NPs were also investigated. The Au(i)-disulfide NPs exhibited similar pH-responsive surface charged states to the Au(i)-Cys complex,^36^ owing to Cys concentrated in the out layer of NPs. The Au(i)-disulfide NPs underwent a decrease in positive charge from 26 mV to 0 mV by increasing pH from 2.2 to 3.0, as well as an increase in negative surface charge from 0 mV to −42 mV over the pH range of 3.0 to 7.4 (Fig. 3A). The isoelectric point at pH 3.0 suggested the disappearance of surface electrostatic repulsion between Au(i) NPs, which impelled many of them to aggregate together and fuse into a large whole at the micro scale as shown in TEM images (Fig. S13A and B†). The increased negative surface charge over the pH range of 3.0 to 7.4 promoted the transformation of the NP aggregates into small lamellar nanostructures (Fig. S13B–F†), in which the size change as shown in TEM images basically coincided with the DLS results (Fig. 3B). The corresponding SAED patterns exhibited a changing molecular arrangement in the NPs from amorphous to crystalline (insets of Fig. S13†), which indicated different aggregated extents of Au(i) NPs at various pH values. To follow their aggregated extent, the UV-vis absorption of Au(i) NPs was measured and showed a hyperchromic shift due to the increasing of the pH to 3.0 caused by the aggregation-induced increase of background scattering, and a hypochromic shift with the pH increase to 7.4 was also observed and could be attributed to the decreased size of NPs (Fig. 3C). Correspondingly, the PL intensity of Au(i)-disulfide NPs reached the maximum at pH 3, and subsequently decreased with the increase of pH value until these NPs were nonemissive at pH 7.4 (Fig. 3D). It was easy to identify pH 3 and pH 7 through the luminescence change by the naked eye when increasing the solution pH values (Fig. S14†). Since the increase of intragastric acidity in the pH range of 3–7 is highly desired in an acid suppress therapy,^5–8^ these unique acidic pH-responsive properties of Au(i) NPs could be potentially used to monitor the intragastric acidity for the assistance of treating gastrointestinal diseases.

**Fig. 4:**
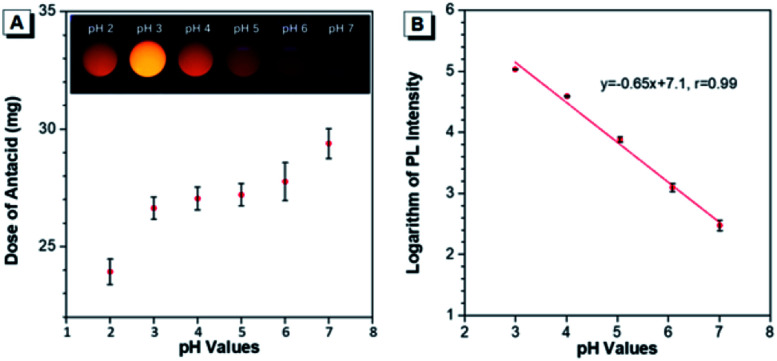
*In vitro* monitoring acidity of the gastric fluid in an acid-suppressed therapy. (A) *In vitro* pH values of the gastric fluid in the presence of Au(i)-disulfide NPs adjusted by different doses of antacid. (B) pH-dependent logarithm of the PL intensity of the Au(i)-disulfide NPs in gastric fluid.

The capability of Au(i)-disulfide NPs to evaluate the intragastric acidity in an acid suppression therapy was first investigated *in vitro*. As one of the commercially available antacid tablets, sodium bicarbonate tablets were widely used to suppress the acidity of gastric fluid. The ultimate pH of gastric fluid was measured by using a pH meter. With the continuously increased dose of antacid (2.7 mg, 3.2 mg, 3.3 mg, 3.8 mg, and 5.5 mg), the pH values of gastric fluid stimulant containing Au(i)-disulfide NPs (3 mL, 1 mg mL^−1^) were adjusted from pH 2 to 3, 4, 5, 6 and 7, respectively (Fig. 4A). The Au(i)-disulfide NPs in the gastric fluid exhibited the strongest luminescence at pH 3 and were nonemissive at pH 7, which could be easily identified by the naked eye (inset of Fig. 4A). There was a good linear relationship between the logarithm of the PL intensity of Au(i)-disulfide NPs and the pH value of the gastric fluid stimulant from 3 to 7 (correlation coefficient, *r* = 0.99) (Fig. 4B).

**Fig. 5:**
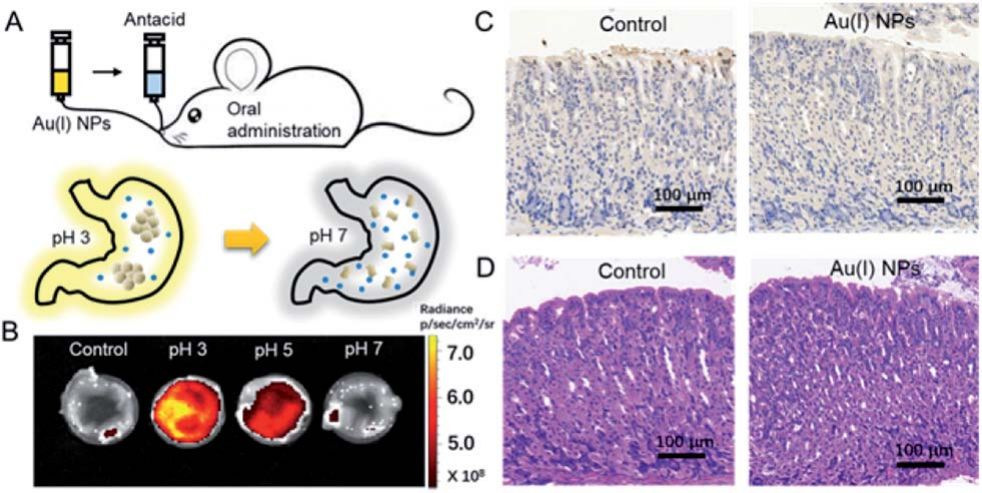
Monitoring intragastric acidity in an acid-suppressed therapy. (A) Schematic illustration of the *in vivo* monitoring gastric acid process by Au(i)-disulfide NPs. (B) Fluorescent images of the stomach of mice cut opened along the greater curvature which was collected after 10 min post administration of Au(i)-disulfide NPs and antacid. The intragastric acidity was suppressed by using different doses of antacid to achieve the pH value of 3, 5, 7. Mice treated with water were used as a control. (C) Section of the mouse stomach from the mice treated with water (control) and Au(i)-disulfide NPs stained with the H&E assay. (D) Sections of the mouse stomach from the mice treated with water (control) and Au(i)-disulfide NPs stained with TUNEL assay.

Finally, the utilization of Au(i)-disulfide NPs for monitoring intragastric acidity in the acid suppress therapy was preliminarily assessed using the mouse model (Fig. 5A). Various doses of antacids were orally administrated to mice for suppressing intragastric acidity. Using water as a control, the intragastric pH value of mice was 1.97. Higher doses of antacids resulted in the decrease of intragastric acidity (7.5 mg for pH 3, 11.4 mg for pH 5 and 15.7 mg for pH 7). The dose of antacids *in vivo* was more than twice that used *in vitro*. Such a dose increase might be associated with the decrease of pH suppression efficacy induced by the continuous secretion of gastric acid in the stomach. The Au(i) NPs were also orally administrated to mice. Given that with the fluorescence signal of these Au(i)-disulfide NPs, it was hard to penetrate the tissue and skin of mice, the stomachs from mice were excised and cut open along the greater curvature for luminescence.

As shown in Fig. 5B, the stomach from mice with intragastric pH 3 displayed the strongest luminescence, while the stomach with intragastric pH 7 was nonemissive. Finally, the gastric toxicity of Au(i)-disulfide NPs was investigated using water as a control. The H&E stains and TUNEL assay indicated that there was no apparent gastric histopathological change or inflammation in the control and experimental groups (Fig. 5C and D). Therefore, in a mouse model, the Au(i)-disulfide NPs with pH-responsive AIE characteristic exhibited potential to monitor intragastric acidity. We believe that with the assistance of optical fibre technology, the utilization of this Au(i) probe would be easy for monitoring intragastric acidity in an acid suppression therapy.

## Conclusions

We have synthesized for the first time a water-stable and biocompatible AIE-active Au(i) complex with pH-responsive characteristics to monitor the intragastric acidity in an acid-suppressed therapy. A pH-responsive moiety, Cys was integrated into the Au(i)-SR complex through disulfide bonds, which were formed by a radical-based mechanism. Cys exiting on the outer layer of the Au(i)-disulfide complex NPs not only provided additional hydrogen bonds to maintain their spherical structure in water, but also induced unique pH-responsive characteristics into NPs. Owing to the change of surface charge over the acidic pH range, the aggregation extents of Au(i)-disulfide NPs varied and led to the obvious luminescence intensity changes at various pH values. These unique pH-responsive AIE characteristics enabled the Au(i)-disulfide NPs to serve as a potential luminescent probe for determining intragastric acidity in an acid-suppressed therapy. This successful example of the utilization of AIE-active Au(i) NPs in monitoring physiological changes increases the applications of luminescent Au(i) complexes, and stimulates the development of the next generation of responsive AIE-active Au(i) complexes with long emission wavelength for directly measuring intragastric acidity by *in vivo* imaging technology.

## Experimental section

### Chemicals

HAuCl_4_·3H_2_O, glutathione (GSH) and *l*-cysteine hydrochloride were obtained from Sigma-Aldrich. Tris(2-carboxy-ethyl)phosphine (TCEP) and other chemicals of at least analytical grade purity were purchased from J&K Scientific Ltd. Ultrapure water prepared by using a Millipore-Q system (≥18 MΩ, Milli-Q, Millipore) was used as the general solvent in this work.

### Materials characterization

The transmission electron microscopy (TEM) images were recorded by using a JEOL JEM-2010 operated at 200 kV. The scanning transmission electron microscopy energy-dispersive spectroscopy (STEM-EDS) images were obtained by using a FEI Titan ETEM equipped with an Oxford energy-dispersive X-ray detector. Samples for Fourier transform infrared (FTIR) spectroscopy were used after vacuum cooling drying and measured by using Bruker FTIR Tensor 37 equipment. PL spectra were taken on a HITACHI F-4500 fluorescence spectrometer. UV-vis absorption spectra were recorded by using a Shimadzu UV-1800 spectrophotometer. PL lifetime measurements were performed with an Edinburgh FLS-920 spectrophotometer with a pulsed light-emitting diode (405 nm) as the excitation source. X-ray photoelectron spectroscopy (XPS) spectra were recorded on a VG Scientific X-ray photoelectron spectrometer (Model ESCALab220i-XL). Dynamic light scattering (DLS) and zeta-potential measurements were taken on a Zetasizer laser light scattering system (NanoZS90, Malvern Instruments Corporation, England). Electrospray ionization mass spectrometry (ESI-MS) was performed by using a DFS high resolution FD-MS (Thermo Fisher Scientific, Bremen, Germany) operating in the positive ion mode. Samples for ESI MS were aqueous solutions without pre-treatments.

### Synthesis of the Au(0)@Au(i) core–shell NCs

The aqueous solutions of GSH (100 mM, 0.3 mL), HAuCl_4_ (16 mM, 1 mL) and ultrapure water (8.7 mL) were mixed together under mild stirring at 25 °C and then incubated at 70 °C for 24 h. Finally, the yellowish solution of the Au(0)@Au(i) core–shell NCs was obtained. Prior to use, the NC solution was stored at 4 °C and had no treatments with any organic solvents.

### Synthesis of Au(i)-disulfide NPs

The obtained Au(0)@Au(i) core–shell NCs and *l*-cysteine hydrochloride aqueous solutions were incubated at 55 °C for 1 h and then cooled to room temperature. The final concentration of *l*-cysteine was 10 mM. Finally, the yellowish transparent solution of Au(i)-disulfide NPs was obtained and could be stored at 4 °C before use.

### *In vitro* study of monitoring intragastric acidity

The commercially available antacid, sodium bicarbonate tablets, were used to suppress the acidity of the gastric fluid simulant (3 mL, initial pH = 1.0) containing Au(i)-disulfide NPs (1 mg mL^−1^). The pH values of the gastric fluid were measured by using a pH meter (Mettler-Toledo, Switzerland) after addition of different amounts of antacid. The PL intensity of Au(i)-disulfide NPs was recorded when the pH value of the gastric fluid was adjusted to 2, 3, 4, 5, 6 and 7, respectively.

### *In vivo* study of monitoring intragastric acidity

Six-week-old male CD-1 mice were purchased from Charles River (China, Beijing). The mice were made to fast for 7 h prior to the experiment but had free access to water. Antacids and Au(i)-disulfide NPs (3 mg) in 0.2 mL water were orally administrated to the mice (four groups, *n* = 3 for each group). Mice treated with 0.2 mL water were tested in parallel as a control. Upon oral administration for 10 minutes, these mice were euthanized and their intragastric acidities were detected immediately by using a microelectrode sensor coupled with a pH meter.

### Gastric toxicity evaluation of Au(i)-disulfide NPs

Six-week-old male CD-1 mice were orally administered with Au(i)-disulfide complexes (3 mg) in 0.2 mL water. Mice administered with 0.2 mL water were used as a control. After the oral administration for 24 h, mice were euthanized and their stomachs were removed for histological analysis, which were evaluated by using the hematoxylin and eosin (H&E) assay and the terminal deoxynucleotidyl transferase-mediated deoxyuridine triphosphate nickend labeling (TUNEL) assay.

## Ethical statement

All animal operations were performed in compliance with the relevant laws and were approved by the guidelines of the Committee on Animal Use and Care of Peking University Shenzhen Graduate School (Shenzhen, China).

## Conflicts of interest

There are no conflicts of interests to declare.

## Supplementary Material

SC-011-D0SC01843K-s001
