# Supplementary material for: pH-Responsive Au(i)-disulfide nanoparticles with tunable aggregation-induced emission for monitoring intragastric acidity
Source: Chem Sci. 2020 Apr 24;11(25):6472–8. doi: 10.1039/d0sc01843k (PMC8159400; doi:10.1039/d0sc01843k)
Supplement: SC-011-D0SC01843K-s001 [file SC-011-D0SC01843K-s001.pdf]

Electronic Supplementary Material (ESI) for Chemical Science.
This journal is © The Royal Society of Chemistry 2020

Supporting Information

pH-responsive Au(I)-disulfide nanoparticles with tunable aggregation-induced emission for monitoring intragastric acidity

Jianxing Wang, Jie Li, Ying Li, Zhijun Zhang, Lei Wang, Dong Wang, Lei Su,* Xueji Zhang,* Ben Zhong Tang**

Table of Contents

Fig. S1 TEM images of Au(0)@Au(I) core-shell NCs S2
Fig. S2 XPS spectra of Au(0)@Au(I) core-shell NCs and Au(I) NPs S3
Fig. S3 Hydrodynamic diameter of Au(I) NPs S4
Fig. S4 Rayleigh scattering of Au(0)@Au(I) core-shell NCs and Au(I) NPs S5
Fig. S5 UV-vis absorption of Au(0)@Au(I) core-shell NCs and Au(I) NPs S6
Fig. S6 The PL stability of Au(I) NPs for a week S7
Fig. S7 PL lifetime decay profiles of Au(I) NPs. S8
Fig. S8 High-revolution ESI-mass spectrum of Au(I) NPs S9
Fig. S9 FT-IR spectra of Au(I) NPs, Au(0)@Au(I) core-shell NCs and Cys S10
Fig. S10 Cleavage of disulfide bonds in Au NPs by TCEP S11
Fig. S11 Hydrodynamic diameter of Au(I) NPs in a water/ethanol mixture S12
Fig. S12 Proposed formation mechanism of Au(I)-disulfide structure. S13
Fig. S13 TEM images of Au(I) NPs in response to different pH values S14
Fig. S14 Digital photos of Au(I) NPs at various solution pH values. S15

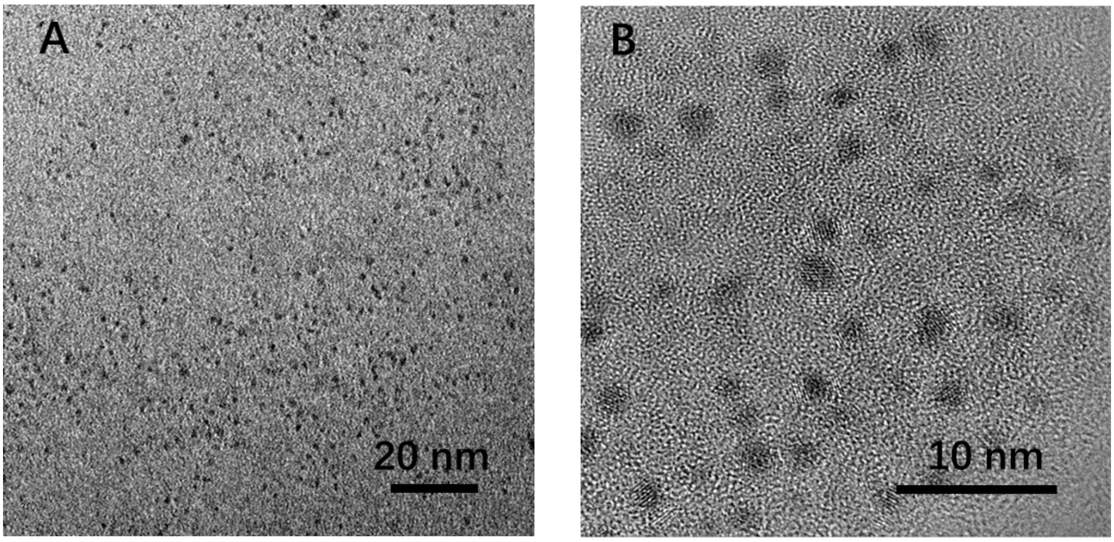

Fig. S1 TEM images of the Au(0)@Au(I) core-shell NCs at different magnifications.

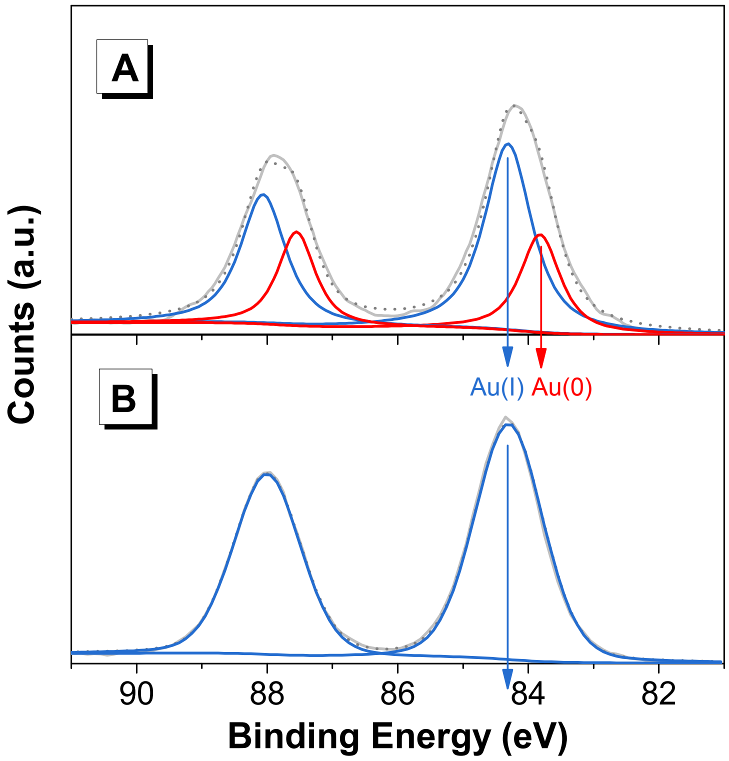

Fig. S2 The Au 4f XPS spectra of (A) Au(0)@Au(I) core-shell NCs and (B) Au(I)-disulfide NPs.

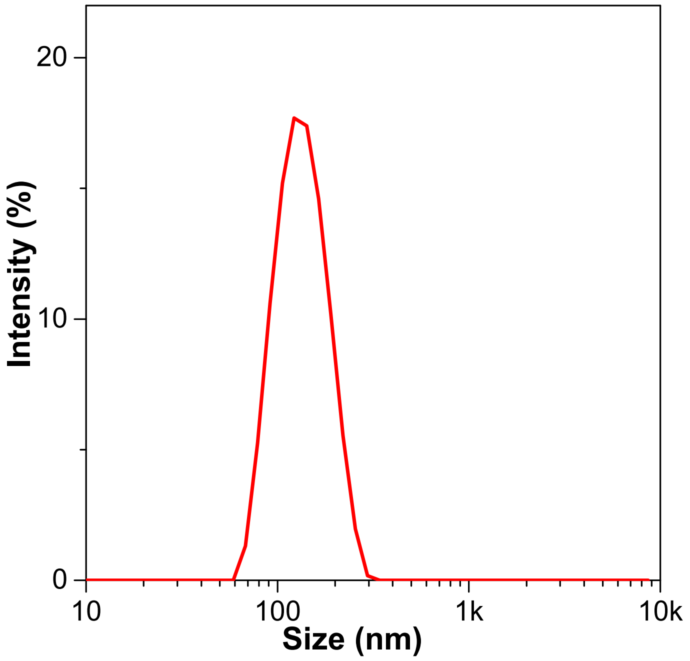

Fig. S3 Hydrodynamic diameter (measured by dynamic light scattering) of Au(I)-disulfide NPs in water.

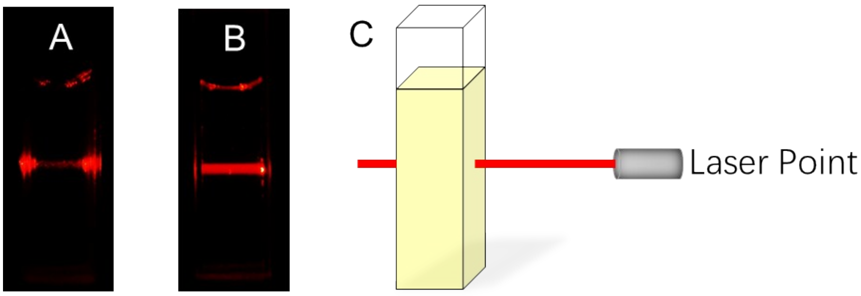

Fig. S4 Digital photos of solutions containing (A) Au(0)@Au(I) core-shell NCs and (B) Au(I)-disulfide NPs. Cuvettes in (A) to (B) were irradiated by the same red laser beam. The test setup for the Rayleigh scattering is shown in (C).

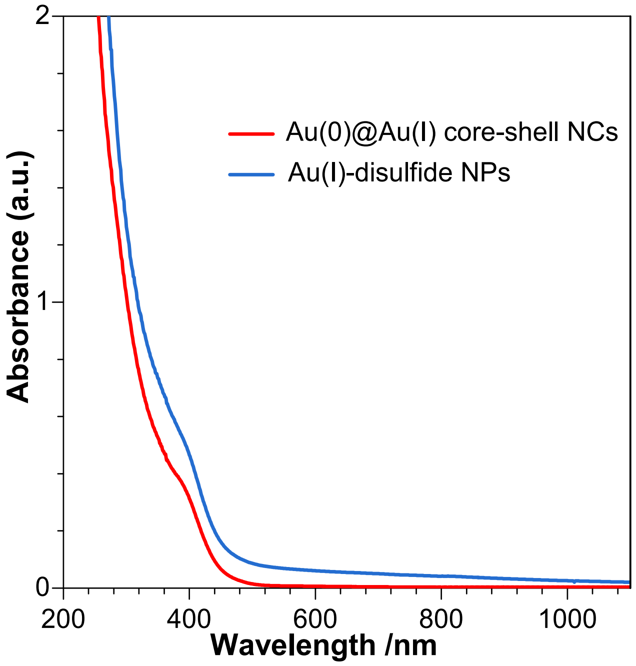

Fig. S5 UV-vis absorption spectra of Au(0)@Au(I) core-shell NCs (red line) and Au(I)-disulfide NPs (blue line).

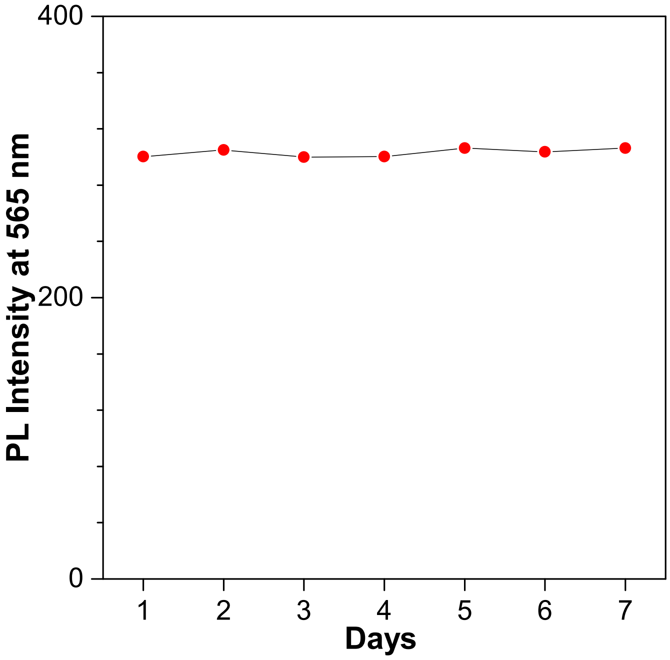

Fig. S6 The PL stability of Au(I)-disulfide NPs for a week.

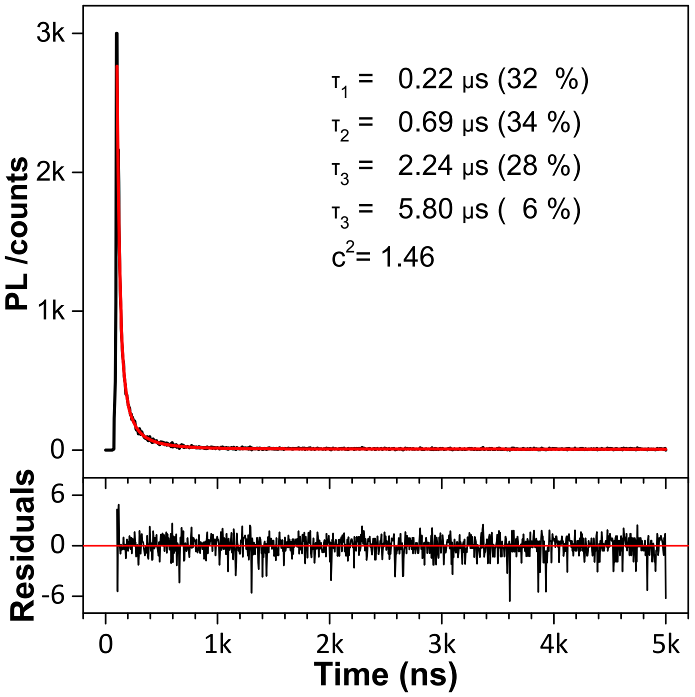

Fig. S7 PL lifetime decay profiles [5.8 μs (6 %), 2.24 μs (28 %), 0.69 μs (34 %), 0.22 μs (32 %)] of Au(I)-disulfide NPs.

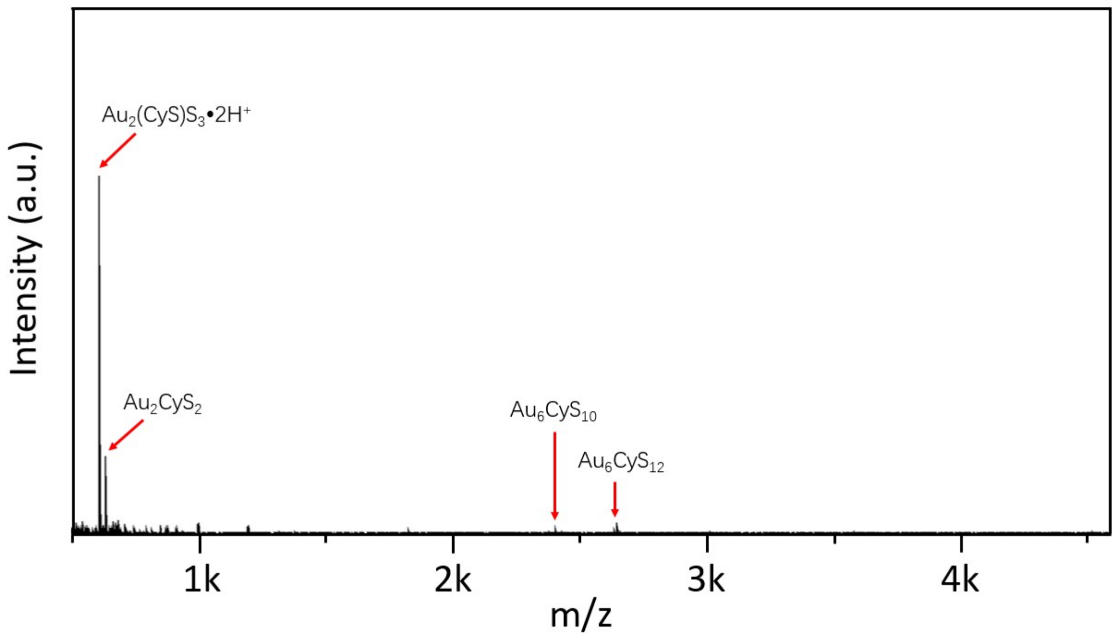

Fig. S8 High-revolution ESI-mass spectrum of Au(I)-disulfide NPs.

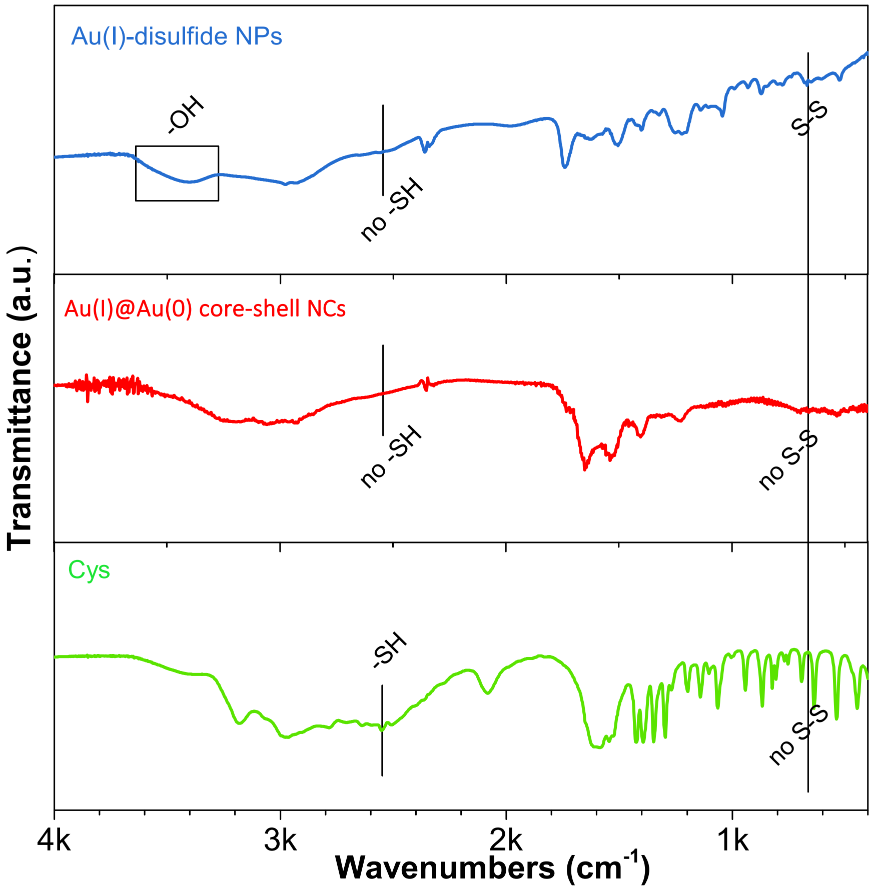

Fig. S9 FT-IR spectra of Au(I)-disulfide NPs, Au(0)@Au(I) core-shell NCs and Cys.

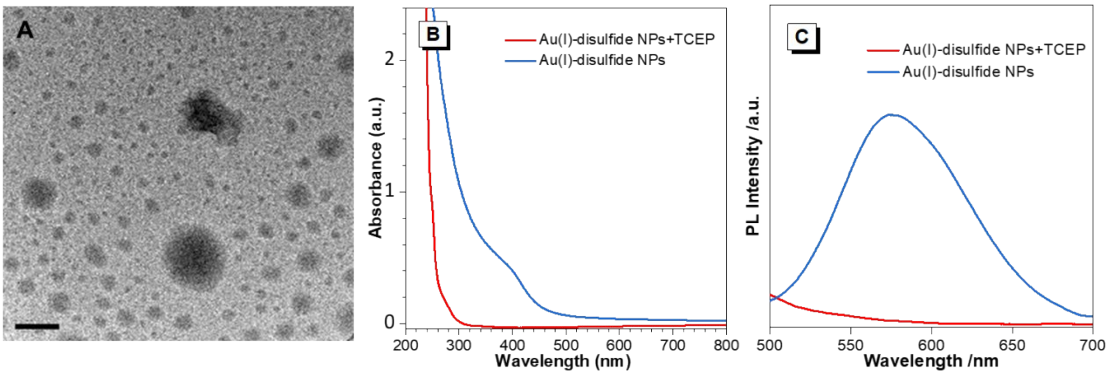

Fig. S10 Cleavage of disulfide bonds in Au(I)-disulfide NPs. (A) TEM images after adding TCEP into the Au(I) nanoparticles. (B) UV-vis absorption spectra of Au(I)-disulfide NPs before (blue line) and after (red line) addition of TCEP. (C) Photoemission spectra of Au(I)-disulfide NPs before (blue line) and after (red line) addition of TCEP.

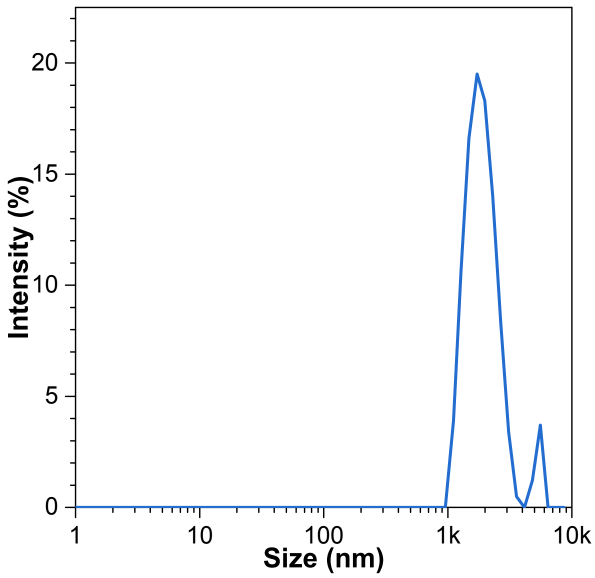

Fig. S11 Hydrodynamic diameter (measured by dynamic light scattering) of Au(I)-disulfide NPs in a water/ethanol mixture with the ethanol fraction (f_e) of 70 %

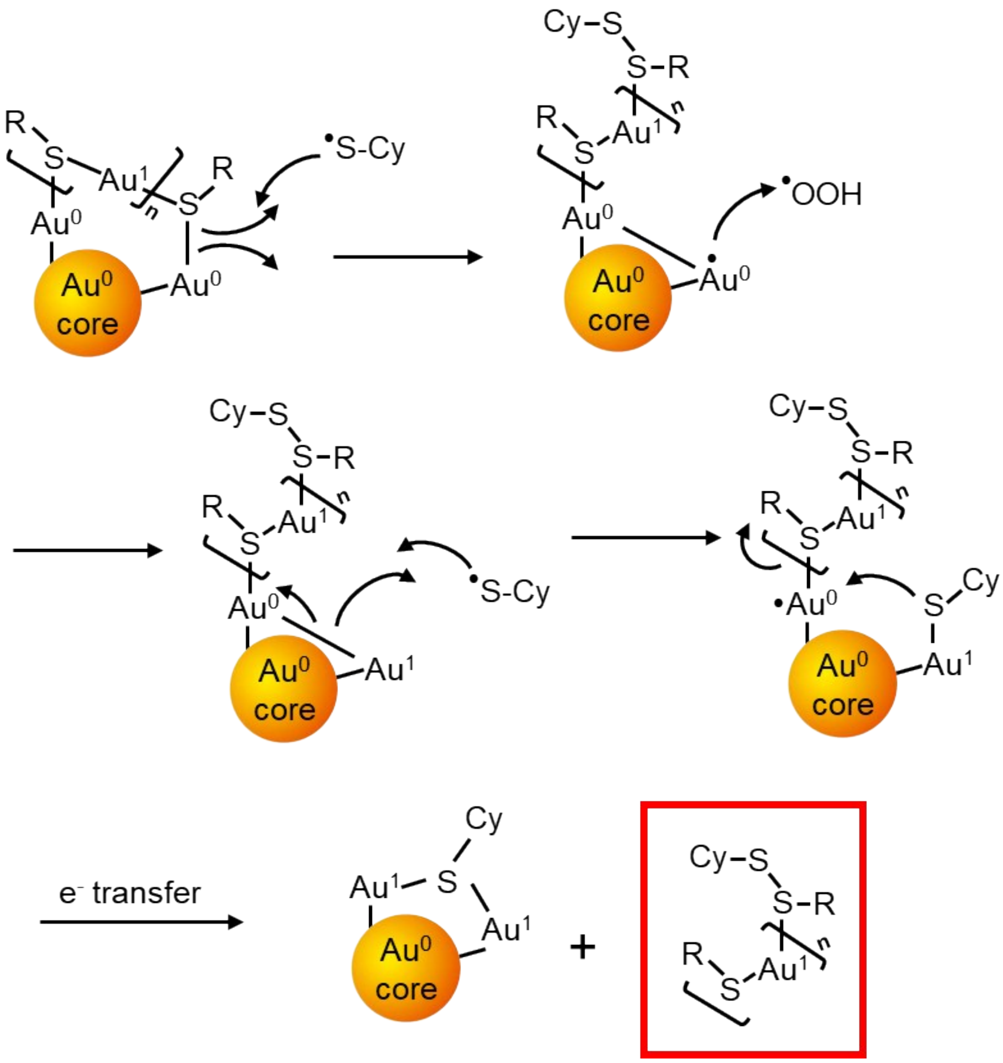

Fig. S12 The proposed radical-based formation mechanism of Au(I)-disulfide structure.

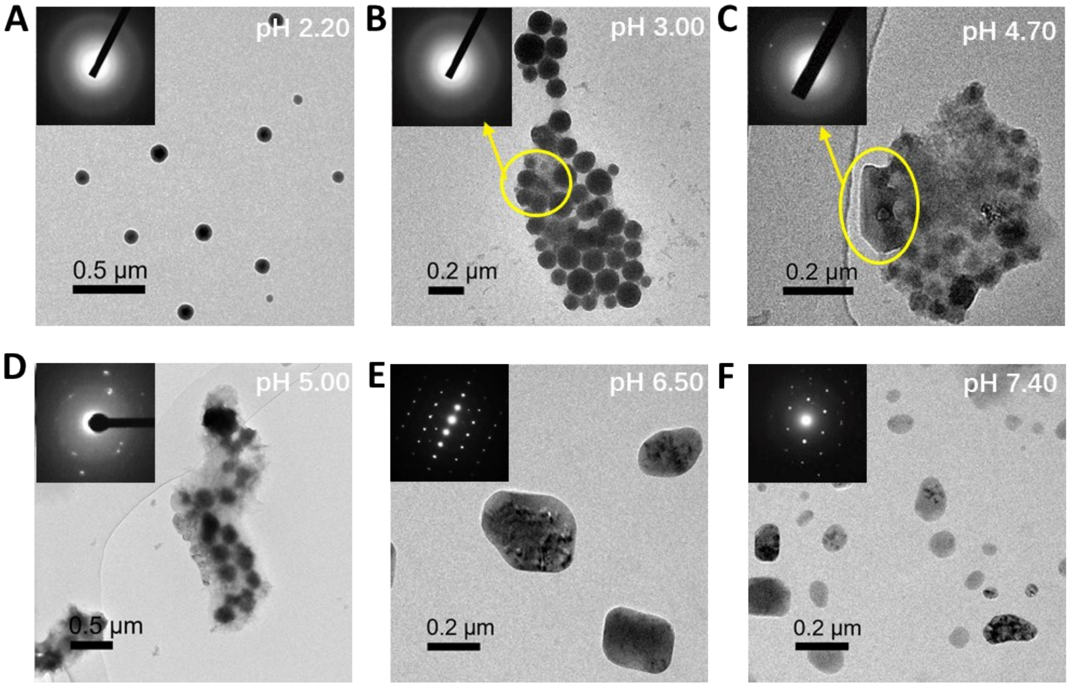

Fig. S13 TEM images of Au(I)-disulfide NPs in response to different pH values. (insets) Corresponding SAED patterns of Au(I)-disulfide NPs in response to different pH values.

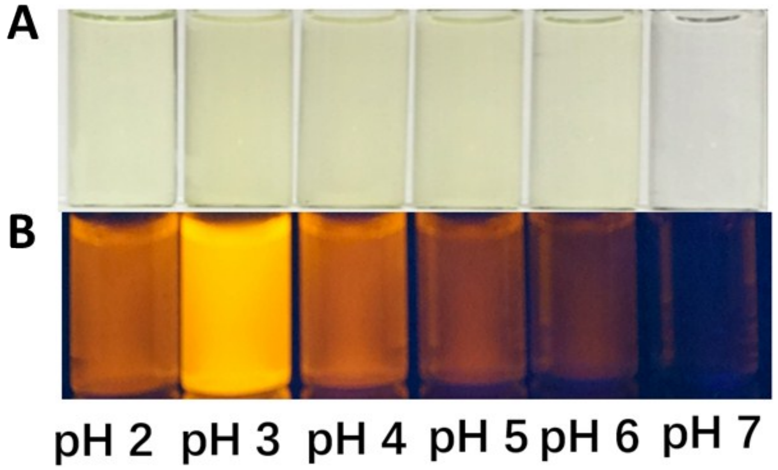

Fig. S14 Digital photos of Au(I)-disulfide NPs under (A) visible light and (B) UV light at various solution pH values.
